# The Effects of Dapagliflozin in Patients With Heart Failure Complicated With Type 2 Diabetes: A Meta-Analysis of Placebo-Controlled Randomized Trials

**DOI:** 10.3389/fcdhc.2021.703937

**Published:** 2021-06-30

**Authors:** Miaobo Zhai, Xin Du, Changmei Liu, Huipu Xu

**Affiliations:** Department of Cardiology, Affiliated Hospital of Binzhou Medical University, Binzhou, China

**Keywords:** dapagliflozin, heart failure, type 2 diabetes, meta-analysis, randomized controlled trial

## Abstract

**Background:**

Cardiovascular disease threatens the health and quality of life of individuals, particularly those with type II diabetes. Recently, some studies have reported the effect of sodium-glucose cotransporter 2 (SGLT2) inhibitors in reducing the rates of hospitalization or urgent visits, resulting in IV therapy for heart failure in patients with type 2 diabetes mellitus (T2DM).

**Methods:**

We did a comprehensive search in electronic databases from inception through July 2020 for randomized-controlled trials, using the keywords “sodium-glucose cotransporter-2 inhibitor”, “dapagliflozin”, “heart failure”, “cardiovascular outcomes”, “major adverse cardiovascular events”, “all-cause mortality”, and “cardiovascular death”. Random-effects summary odds ratios (OR) were constructed using M-L heterogeneity model.

**Results:**

Five trials with 5,252 patients were ultimately included. The incidence of hospitalization for heart failure (HHF) (n=4, OR=0.74; 95% CI, 0.61 to 0.88; I^2^ = 0%) and all-cause mortality (ACM, n=4, OR=0.76; 95% CI, 0.66 to 0.94; I^2^ = 0%); was reduced by dapagliflozin, respectively, in all heart failure patients, without obvious heterogeneity. The incidence of cardiovascular death in dapagliflozin was lower than that in placebo without statistically significant (CVD, n=5, OR=0.84; 95% CI, 0.69 to 1.03; I^2^ = 0%). In HFrEF subgroup, dapagliflozin was associated with a reduced incidence of hospitalization for heart failure (n=4, OR=0.74; 95% CI, 0.60 to 0.91; I^2^ = 0%), cardiovascular death (n=4, OR=0.72; 95% CI, 0.58 to 0.91; I^2^ = 8%), and all-cause mortality (n=3, OR=0.70; 95% CI, 0.50 to 0.99; I^2^ = 43%) without significant heterogeneity. In contrast, in the HFpEF subgroup, there was no difference in the incidence of cardiovascular death (n=2, OR=1.45; 95% CI, 0.95 to 2.22; I^2^ = 0%) and all-cause mortality (n=2, OR=1.04; 95% CI, 0.76 to 1.43; I^2^ = 0%) between dapagliflozin and placebo.

**Conclusion:**

In our study, dapagliflozin performed a statistical reduction in the rate of heart failure hospitalization, cardiovascular death, and all-cause mortality in patients with HFrEF and diabetes. However, in the HFpEF subgroup, dapagliflozin did not show a significant cardiovascular protective effect.

## Introduction

Heart failure has been a major reason of demise and threat of around 26 million population around the globe (1). Unfortunately, more than half of these patients died within 5 years after their first admission, and even 2% to 17% cannot survive within a year. The causes of high fatality rate and short survival period of heart failure are complex, among which, the most important one is type 2 diabetes. The high risk of heart failure in diabetic patients is not associated with coronary heart disease, and there are limited data to guide heart failure prevention therapy (2, 3). However, although these two malfunctions can be well treated independently, finding a remedy to heart failure involving with type 2 diabetes is still a challenge.

Sodium–glucose cotransporter-2(SGLT-2) inhibitors are a class of hypoglycemic agents with novel cardiovascular benefits. SGLT-2 inhibitors block the sodium-glucose co-transporter in the proximal convoluted tubule of the kidney, thereby halting glucose reabsorption and reducing blood glucose by increasing the excretion of urinary glucose (4). The current studies also illustrated that SGLT-2 inhibitors could reduce blood pressure and body weight, which might lead to cardiovascular benefits. In contrast, some studies reported that SGLT-2 inhibitors might raise the risk of ketoacidosis and urinary tract/genital mycotic infection in patients with diabetes (5). In the current studies, other SGLT2 inhibitors have shown beneficial effects regarding hospitalization of patients with heart failure, with or without diabetes (6–8). SGLT2 inhibitors also demonstrated delay in renal dysfunction (6, 8–11). Dapagliflozin, one of the SGLT-2 inhibitors classes, is widely used in clinics. At present, some large RCTs about dapaflifolzin, such as DAPA-CKD, DAPA-HF, and Declar-Timi 58, have been published. In these studies, dapagliflozin showed significant cardiovascular benefits. To comprehensively analyze the effects of dapagliflozin in cardiovascular outcomes and to give clinician appropriate advice, we conducted this systematic review and meta-analysis.

## Methods

### Selection Criteria and Data Extraction

This meta-analysis was conducted according to the Preferred Reporting Items for Systematic reviews and Meta-Analyses (PRISMA) guidelines (12). We performed a comprehensive search in PubMed, EMBase, Web of Science, and Cochrane Library (up to July 2020), the related searching keywords were as follows: “sodium–glucose cotransporter-2 inhibitor”, “dapagliflozin”, “heart failure”, “cardiovascular outcomes”, “major adverse cardiovascular events”, “all-cause mortality”, and “cardiovascular death”. We also searched local database (CNKI, VIP, CBM, and WanFang DATA) and Grey literature database like OPENGREY. The detailed search algorithm are as follows: (((((dapagliflozin [Title/Abstract]) OR (Farxiga [Title/Abstract])) OR (Forxiga [Title/Abstract])) OR (Sodium-Glucose Transporter 2 Inhibitors [MeSH Terms])) AND ((((((((heart failure [Title/Abstract]) OR (HF [Title/Abstract])) OR (HFrEF [Title/Abstract])) OR (HFpEF [Title/Abstract])) OR (cardiovascular outcomes [Title/Abstract])) OR (major adverse cardiovascular events [Title/Abstract])) OR (all-cause mortality [Title/Abstract])) OR (cardiovascular death [Title/Abstract]))) AND (((randomized controlled trial) OR (RCT)) OR (placebo control)). The inclusion criteria were the following: 1) only dapagliflozin was used as an intervention; 2) randomized-controlled trials published in English or Chinese; 3) patients with heart failures (both HFrEF and HFpEF) and T2DM; 4) studies that reported at least one of our selected cardiovascular outcomes. Our primary outcomes included hospitalization for heart failure (HHF) and cardiovascular death (CVD). The secondary outcomes included all-cause mortality (ACM). The exclusion criteria were as follows: 1) not RCTs; 2) patients on other SGLT-2 inhibitors treatment; 3) study with incomplete data reporting. Two investigators (ZM and DX) selected the studies and extracted relevant data about trial characteristics and our selected outcomes independently. Discussion was performed for inconsistencies. If one trial reported multiple data, only the most complete one was used. When the placebo was converted to an active comparator during the extended trial period, only the placebo part was included. Quality assessment was completed according to GRADE approach and Cochrane tool RoB 2 by two independent investigators. The number of primary and secondary outcomes in both dapagliflozin and placebo group was extracted from the included studies. Basic data extracted from study included trial design (year, dapagliflozin dose, and follow up) and patient characteristics (age, male, hypertension, baseline serum creatinine level, baseline estimated glomerular filtration rate [eGFR], and base treatment).

### Statistical Analysis

Intention-to-treat analysis was used to analyze the outcomes. For dichotomous data, we calculated odds ratio (OR) with 95% confidence intervals (CIs). For the potential heterogeneity between the included trials, we used random-effects model for data analysis. I^2^ statistic value was used to evaluate the statistical heterogeneity (define as<25%=low heterogeneity, 25% to 50%=moderate heterogeneity, and >50%=high heterogeneity) (13). Sensitivity analysis was completed to identify the influence of each trials and the potential source of heterogeneity. The risk of publication bias was detected by Begg’s test and Egger’s test. All statistical analyses were performed using RevMan (version 5.4; Cochrane Collaboration, Oxford, UK), RoB-2 tool, and Stata (version 12.0; Stata Corporation, College Station, TX).

## Results

**Figure 1:**
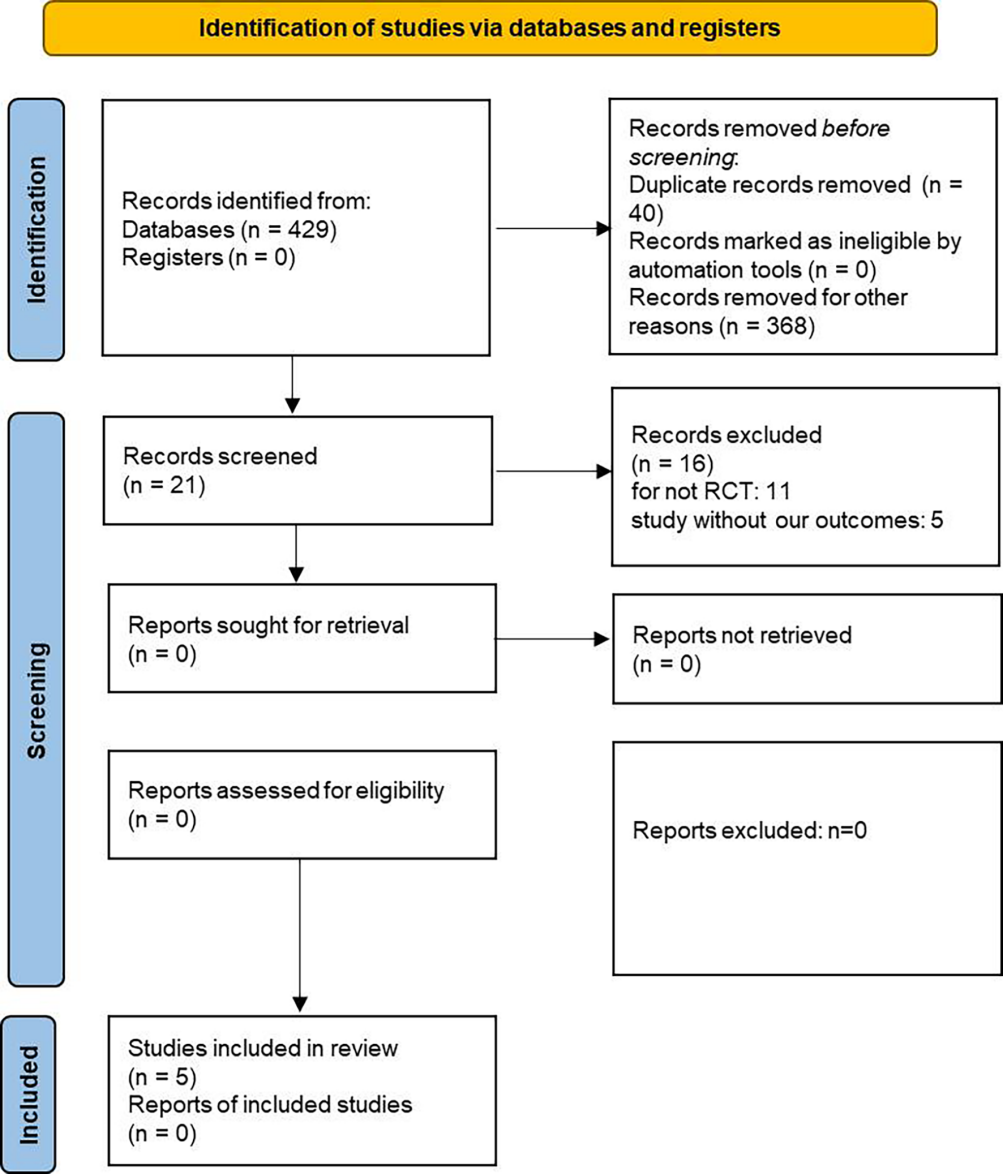
Flow diagram for data searching and selecting process.

**Figure 2:**
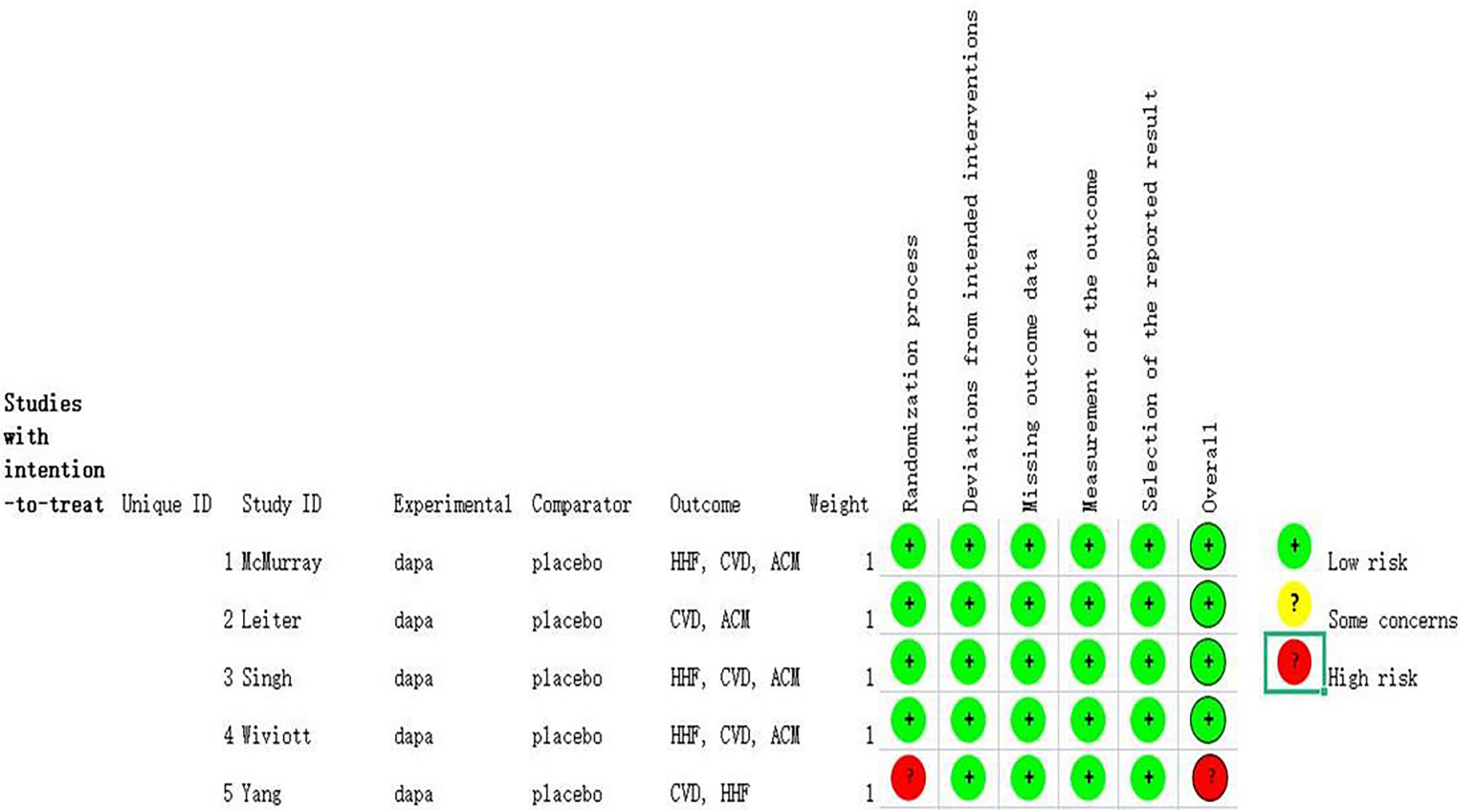
Risk of bias assessed by Cochrane tools RoB 2.

The details about searching and selecting process for the RCTs in this study are displayed in **Figure 1**. Five trials (14–18) with a total of 5,252 patients ultimately met the criteria. Wiviott’s study included a total of 17,169 participants, in which only 1,987 participants were eligible for inclusion and were included in our study (19). McMurray’s study included 4,744 patients with heart failure, and 2,139 of whom were complicated with diabetes. Only the 2,319 participants with coexisting heart failure and diabetes in McMurray’s study were included in our analysis. The details of the included studies are shown in **Table 1**. Two of the studies were large RCTs with more than 1,000 participants (15, 17), whereas the other studies all had less than 1,000 (14, 16, 18). Four studies reported hospitalization for heart failure (15–18). All five studies reported cardiovascular death outcome. Except for the Yang’s study, four studies reported all-cause mortality. All the studies used dapagliflozin 10 mg/d as the only intervention factor and placebo as control. The quality assessment according to GRADE approach was shown in **Table 2**. The risk of bias was assessed by Cochrane tools RoB 2 (**Figure 2**).

**Table 1:** Demographics and baseline characteristics of the included study.

Study	No. of participant		Mean age		Follow up	Trial design	Blind method	Outcomes	Type of HF
	dapa	Placebo	dapa	placebo					
Leiter,2014	482	483	63.9	63.6	52w	placebo- controlled RCT	Double-blind	CVD and ACM	HFpEF
McMurray,2019	2373	2371	66.2	66.5	18.2m	placebo- controlled RCT	Double-blind	HHF, CVD, and ACM	HFrEF
Singh,2020	28	28	67.1		1y	placebo- controlled RCT	Double-blind	HHF, CVD, and ACM	HFrEF
Wiviott,2019	980	1007	63.9	64.0	4.2y	placebo- controlled RCT	Double-blind	HHF, CVD, and ACM	HFpEF and HFrEF
YangZhen,2019	53	52	65.18	66.31	6m	placebo- controlled RCT	N/A	HHF and CVD	HFrEF
Study	Age (year)	Female no.(%)	LVEF(%)	Hypertension (%)	eGFR	Fasting plasma glucose (mmol/l)	Treatment (%)		
							ACEI or ARB	Beta-blocker	Lipid-reducing
Leiter	63.7	318 (33.1)	N	93	N	9.12	83.5	73.8	84.3
McMurray	66.4	1109 (23.4)	31.1	N	65.75	N	83.6	96.1	N
Singh	N	N	N	N	N	N	N	N	N
Wiviott	64.0	669 (33.7)	N	95	85.25	N	86.2	80.8	82.2
YangZhen	65.8	47 (44.8)	N	63.8	N	7.76	88.6	83.8	92.4

**Table 2:** Result of grade quality assessment.

Group	No. of study	Design	Risk of bias	Inconsistency	Indirectness	Imprecision	Quality	Importance
Heart failure hospitalization	4	RCT	no serious	no serious	no serious	no serious	high	critical
Cardiovascular death	5	RCT	no serious	no serious	no serious	no serious	high	critical
All-cause mortality	4	RCT	no serious	no serious	no serious	no serious	high	critical

### Effect of Dapagliflozin on the Hospitalization for Heart Failure (HHF)

**Figure 3:**
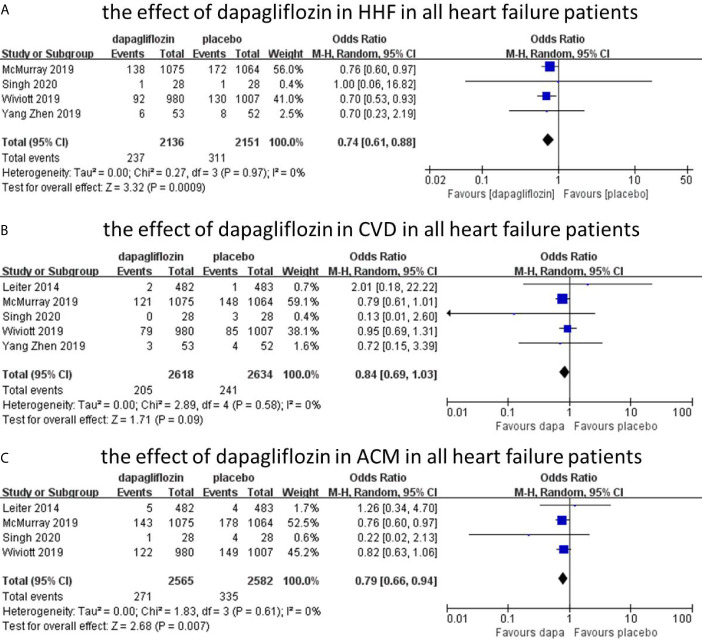
The effect of dapagliflozin in HHF in all heart failure patients **(A)**. The effect of dapagliflozin in CVD in all heart failure patients **(B)**. The effect of dapagliflozin in ACM in all heart failure patients **(C)**.

The hospitalization for heart failure was reported in four studies with 4,287 patients, and the overall incidence was 12.8% (dapagliflozin, 11.1%; control, 14.5%).The incidence of HHF was reduced by dapagliflozin (n = 4, OR = 0.74; 95% CI, 0.61–0.88; I^2^ = 0%; **Figure 3A**). The sensitivity analysis was not conducted because of low heterogeneity (I^2^ = 0%). The assessment of publication bias (Begg’s test, P =1.0, Egger’s test, p=0.870) showed no significant risk for bias.

### Effect of Dapagliflozin on the Cardiovascular Death (CVD)

Five studies reported cardiovascular death outcome with 5,252 patients. The overall incidence of cardiovascular death was 8.5%. Among them, the incidence was 7.8% in dapagliflozin group and 9.1% in placebo group. The incidence of dapagliflozin group was lower than placebo, but it was not statistically different between the two groups (n=5, OR=0.84; 95% CI, 0.69 to 1.03; I^2^ = 0%; **Figure 3B**). No significant publication bias was detected by Begg’s test (p=0.806) and Egger’s test (p=0.747).

### Effect of Dapagliflozin on All-Cause Mortality (ACM)

Four studies reported all-cause mortality, with 5,147 patients. The overall incidence of all-cause mortality was 11.8%, with 10.6% in dapagliflozin group and 13.0% in control group. The incidence of all-cause mortality was reduced by dapagliflozin (n=4, OR=0.76; 95% CI, 0.66 to 0.94; I^2^ = 0%; **Figure 3C**). No evidence of publication bias was detected in the analysis (Begg’s test, p = 1.0; Egger’s test, p = 0.754).

### Subgroup Analysis

**Figure 4:**
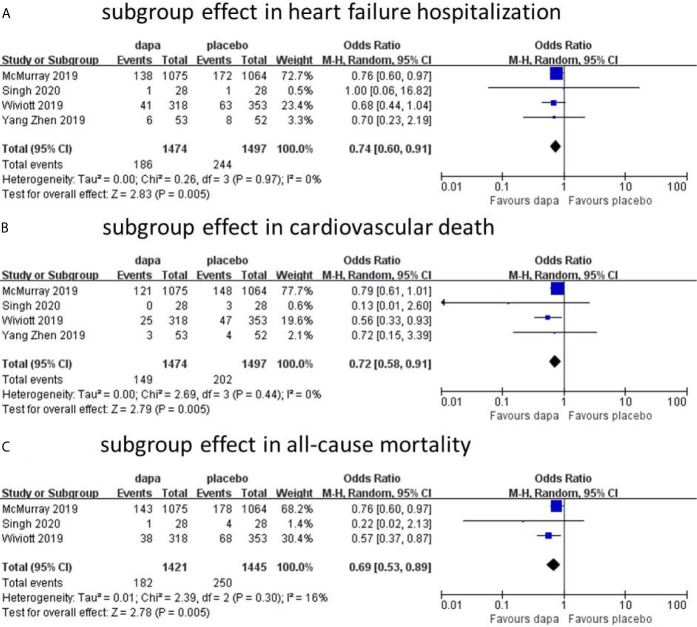
Subgroup effect in heart failure hospitalization **(A)**. Subgroup effect in cardiovascular death **(B)**. Subgroup effect in all-cause mortality **(C)**.

**Figure 5:**
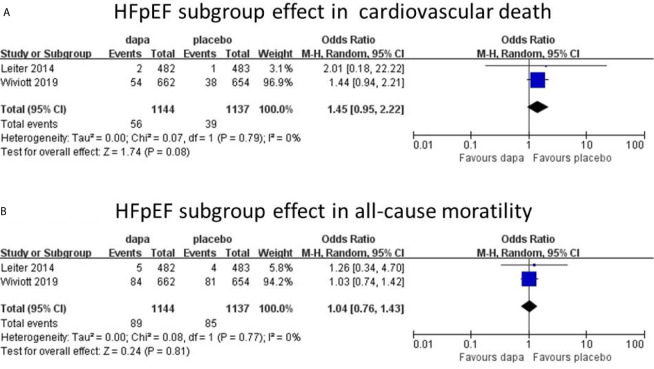
HFpEF subgroup effect in cardiovascular death **(A)**. HFpEF subgroup effect in all-cause mortality **(B)**.

In all heart failure patients, Dapagliflozin had shown some benefits in hospitalization for heart failure and all-cause mortality with statistical significance. In cardiovascular death group, the incidence of CVD in dapagliflozin group was lower than placebo group without statistical significance. However, heart failure was not stratified during the data analyses. To further analyze the cardiovascular protective effect of dapagliflozin in different types of heart failure, we performed a subgroup analysis based on stratified left ventricular ejection fraction (LVEF) value. In subgroup analysis, heart failure with reduced ejection fraction (HFrEF) and heart failure with preserved ejection fraction (HFpEF) were classified according to left ventricular ejection fraction (defined as HFrEF: LVEF<45%, HFpEF: LVEF≥45%). In HFrEF subgroup, the incidence of hospitalization for heart failure, cardiovascular death, and all-cause mortality was reduced by dapagliflozin, respectively (n=4, OR=0.74; 95% CI, 0.60 to 0.91; I^2^ = 0%; **Figure 4A**; n=4, OR=0.72; 95% CI, 0.58 to 0.91; I^2^ = 0%; **Figure 4B**; and n=3, OR=0.69; 95% CI, 0.53 to 0.89; I^2^ = 16%; **Figure 4C**). To explain the heterogeneity in all-cause mortality (I^2^ = 16%), we did a sensitivity analysis and showed that all included trials are almost consistent with the 95% confidence interval. In contrast, in the HFpEF subgroup, the incidence of cardiovascular death (n=2, OR=1.45; 95% CI, 0.95 to 2.22; I^2^ = 0%; **Figure 5A**) and all-cause mortality (n=2, OR=1.04; 95% CI, 0.76 to 1.43; I^2^ = 0%; **Figure 5B**) in dapagliflozin group showed no superiority over placebo. Because of lack of data, about the effect of dapagliflozin in patients with HFpEF and diabetes, the subgroup analysis about heart failure hospitalization in HFpEF was not conducted.

### Trial Sequential Analysis

To confirm the pooled effect size of dapagliflozin as a true estimated effect, a sample size of 1,538 for heart failure hospitalizations, 9,773 for cardiovascular deaths, and 4,791 for all-cause deaths were required (20). As indicated by the trial sequential analysis, future trials will need to add approximately 1,916 samples of CVD. However, the sample size of HHF (4287 *vs* 1538) or ACM (5147 *vs* 4791) is enough for the power of estimated effect.

## Discussion

This is a systematic review and meta-analysis with five RCTs enrolling 5,252 patients treated by dapagliflozin in dose 10 mg/day. In all heart failure patients (non-stratified by LVEF), the incidence of hospitalization for heart failure and all-cause mortality was reduced by dapagliflozin with statistical significance. In the cardiovascular death group, the incidence of CVD in dapagliflozin group was lower than the placebo group, but the result was not significant statistically. In the HFrEF subgroup, dapagliflozin showed cardioprotective effects in all three group. However, in the HFpEF subgroup, dapagliflozin showed no superiority over placebo in cardiovascular death and all-cause mortality. To our knowledge, our study is the first meta-analysis concern about the clinical outcomes of dapagliflozin in hospitalization for heart failure, cardiovascular death, and all-cause mortality in patients with heart failure and type 2 diabetes.

Patients with diabetes are at higher risk for adverse outcomes from heart failure (21). Although great progress has been made in the treatment of heart failure in recent years, the long-term prognosis of patients with heart failure has lagged behind. The causes of high fatality rate and short survival period of heart failure are complex, among which the most important one is type 2 diabetes (22–26). In the same way, the prevalence of type 2 diabetes is higher in patients with heart failure, with some reports of more than 40% (27). The prevalence of heart failure in patients with diabetes is between 9% and 22%, which is four times higher than that of general population (28), and the data are even higher in elderly patients (age, >60 years) (29–31). Despite the treatments available for heart failure and type 2 diabetes, respectively, strategies for patients with heart failure complicated type 2 diabetes are incomplete (32). SGLT2i is a class of glucose-lowering agent that can significantly improve cardiovascular outcomes in patients with type 2 diabetes (19). In our observation, dapagliflozin could reduce heart failure hospitalization and all-cause mortality in patients with type 2 diabetes mellitus and heart failure, but showed no statistical difference in cardiovascular death. We also conducted publication bias assessment and sensitivity analysis to detect the power of the effect of dapagliflozin in cardiovascular outcomes. To comprehensively analyze the effect of dapagliflozin in patients with different left ventricular ejection fraction value, we performed a subgroup analysis based on LVEF stratification. In HFrEF subgroup, compared with the placebo, the heart failure hospitalization (n=4, OR=0.74; 95% CI, 0.60 to 0.91; I^2^ = 0%), cardiovascular death (n=4, OR=0.72; 95% CI, 0.58 to 0.91; I^2^ = 0%), and all-cause mortality (n=3, OR=0.69; 95% CI, 0.53 to 0.89; I^2^ = 16%) were reduced in the dapagliflozin group without obvious heterogeneity. These results were not completely consistent with those in the non-stratified. However, in the HFpEF subgroup, dapagliflozin did not show superior cardiovascular effect over placebo in cardiovascular death and all-cause mortality. Because of the lack of included study for heart failure hospitalization in the HFpEF subgroup, no subgroup analysis was performed for this section. Currently, some ongoing studies, like DELIVER study and EMPEROR-PRESEVED, are assessing the effects of SGLT-2i, which will provide more information about the issue of SGLT-2i in patients with coexisting heart failure of preserved ejection fraction and type II diabetes (33, 34).

Lots of meta-analysis concerning empagliflozin and canagliflozin have been published. However, there is no meta-analysis about dapagliflozin in patients with coexisting heart failure and diabetes available. In this study, we assessed the cardiovascular benefits of dapagliflozin in patients who either have heart failure or type 2 diabetes. Of note, our results were not completely consistent with the previous trials. For example, the cardiovascular benefits of dapagliflozin were consistent with EMPA-REG OUTCOME and CANVAS program in HFrEF subgroup. However, in the HFpEF subgroup, no cardiovascular benefit was observed in our study, which is not consistent with the EMPA-REG OUTCOME. Moreover, we first conducted trial sequential analysis and showed that, if future trials are to achieve the same event rate, the hospitalization for heart failure and all-cause mortality do not need to recruit additional participants to provide future meta-analyses with the power to confirm the cardiovascular benefits. However, the cardiovascular death would require an expansion of the participants to stabilize. All these data suggest that dapagliflozin may be an effective clinical treatment to reduce hospitalization and mortality in patients with heart failure and type 2 diabetes.

Our study confirms the cardioprotective effect of dapagliflozin on patients with heart failure, particularly heart failure with reduced ejection fraction and type 2 diabetes. The mechanism of dapagliflozin in cardiovascular protecting is complicated, and, in some aspect, remains unclear. The mechanism may involve improvement of ventricular loading conditions, improvement of cardiac metabolism and bioenergetics, Na+/H+ exchange, sugar and lipid metabolism, circulatory load, cardiovascular system, and other aspects (35). Unlike other glucose-lowering agents, SGLT2 inhibitors offer an insulin-independent mechanism to lower blood glucose by increasing urinary glucose excretion (36). The recent meta-analyses and clinical evidence have indicated that SGLT-2 inhibitors are effective in lowering blood pressure and body weight (37). To date, it is generally accepted that the main mechanism of dapagliflozin in protection of heart failure is improving ventricular loading conditions and reducing preload *via* diuretic and natriuretic (38, 39). It is worth noting that natriuretic also associated with renal protection by stimulating tubuloglomerular feedback. SGLT-2 inhibitors can also reduce plasma volume by selectively reducing interstitial fluid, followed by reduced preload, decreased arterial stiffness, and blood pressure, which, in turn, would reduce afterload, leading to an improved coronary circulation (40). In ion transporting, some scholars proposed that SGLT-2 inhibitors may inhibit Na+/H+ exchanger (NHE) 1 isoform (41, 42). SGLT-2 inhibitors could increase mitochondrial calcium levels and reduce cytoplasmic sodium and calcium levels by inhibiting NHE (43). SGLT-2 inhibitors can also downregulate the activity of Na+/H+ exchanger 3 in the proximal tubule, through which, promote natriuresis and reduce heart failure (44). In addition, SGLT2 inhibitors also reduce total fat mass and regional adipose tissue distribution, both of them are associated with heart failure (45). In addition to the mechanism described above, the effects of SGLT2 inhibitors on myocardial metabolism, fibrosis, adipokines, and vascular function have also been proposed (35, 40, 41, 46, 47). Moreover, as provocative animal studies of SGLT-2 inhibitors have shown reductions in oxidative stress, improvement in endothelial function and neurohormonal modulation, and anti-inflammatory effects (48, 49), the mechanisms beyond glucose lowering or diuresis may partly explain the reduction in heart failure events (48). Combined with our results, the described mechanism may partially explain the cardioprotective effect of dapagliflozin in patients with heart failure. However, the mechanism of dapagliflozin is still unclean and needs to be revealed by further study.

In this meta-analysis, two of the included trials were large RCTs with more than 1,000 participants, whereas the other four trials involved less than 1,000 participants. The satisfaction is that dapagliflozin was the only intervention factor in all studies, whereas placebo was used as control. The dose of dapagliflozin was 10 mg/day, and the mean follow-up time was 19.43 months. We also found a trial in which doses of 5, 25, and 10 mg/day were used as intervention (50). In this study, dapagliflozin showed a dose-dependent effect on reduction in fasting serum glucose. Considering its small sample size, it was not analyzed as a subgroup. It must be acknowledged, however, that the optimal dose for dapagliflozin remains unclear and should be the focus in future studies.

The main strength of this analysis is providing hypothesis-generating findings for future RCTs to test the well-defined hypothetic clinical outcomes of dapagliflozin in heart failure. It is important to note that the observed effect of dapagliflozin in HFpEF is not consistent with the EMPA-REG OUTCOME study. This result needs to be further confirmed by the addition of a large RCT. However, our findings should be interpreted with caution, owing to some potential limitations. First, the characteristics of the patients at baseline in some studies were roughly reported, which made it difficult to analyze the impact of potential cardiovascular complications (such as valvular heart disease, etc.) and basic treatment. Second, the inclusion of small-scale RCTs in studies may affect the results with uncertainty. Third, although we used statistical methods, such as Begg’s test and Egger’s test, to evaluate publication bias and searched the gray literature database, the potential effect of unpublished data could not be completely ruled out. Fourth, as for the dose of dapagliflozin, we chose the usual dose of 10 mg/day, but the effect of dose change on the results needs further experimental verification. Last, but not the least, there are few RCTs included in the study, and most of the participants are from two large RCTs. Although these two studies have reached high quality through Cochrane and GRADE quality assessments, the potential impact on the final results is still difficult to determine.

## Conclusion

SGLT-2 inhibitors are first class glucose-lowering drugs, which have been shown to reduce hospitalization rates for heart failure in diabetic patients (32). In our study, dapagliflozin was associated with a statistical reduction in the heart failure hospitalization, cardiovascular death, and all-cause mortality in patients with heart failure with reduced ejection fraction and diabetes. This result was consistent with the EMPA-REG OUTCOME study. However, in the HFpEF subgroup, dapagliflozin did not show a significant cardiovascular protective effect. The underlined mechanism needs further research. To sum up, our study found that dapagliflozin can reduce the heart failure hospitalization cardiovascular death and all-cause mortality in patients with HFrEF and diabetes, but has no cardiovascular benefits in patients with HFpEF. The result indicates that dapagliflozin may be considered as an important component of treatment for patients with heart failure and diabetes, particularly in patients with HFrEF. The role of dapagliflozin in cardiovascular disease will be updated with the inclusion of more high-quality clinical RCTs.

## Data Availability Statement

The original contributions presented in the study are included in the article/supplementary material. Further inquiries can be directed to the corresponding author.

## Author Contributions

All authors listed have made a substantial, direct, and intellectual contribution to the work and approved it for publication.

## Conflict of Interest

The authors declare that the research was conducted in the absence of any commercial or financial relationships that could be construed as a potential conflict of interest.
